# Age-Dependent Effect of Myostatin Blockade in the *mdx* Mouse Model of Duchenne Muscular Dystrophy (DMD)

**DOI:** 10.3390/muscles5030058

**Published:** 2026-08-20

**Authors:** Sasha Bogdanovich, Emidio E. Pistilli, Tejvir S. Khurana

**Affiliations:** 1 Department of Physiology, Pennsylvania Muscle Institute, Perelman School of Medicine, University of Pennsylvania, 415 Curie Boulevard, Philadelphia, PA 19104, USA; sashab@pennmedicine.upenn.edu (S.B.); epistilli2@hsc.wvu.edu (E.E.P.); 2 School of Medicine, West Virginia University, Morgantown, WV 26506, USA

**Keywords:** myostatin blockade, Activin, *mdx*, Duchenne Muscular Dystrophy, young, neonatal improvement, Multiparametric Muscle Improvement Score, MMIS

## Abstract

Myostatin (GDF8) is a member of the transforming growth factor-β (TGF-β) superfamily. Myostatin gene mutations or inhibition of the Myostatin/Activin pathway results in increased skeletal muscle mass, demonstrating its role as a negative regulator of skeletal muscle. Myostatin blockade is a promising strategy for increasing muscle mass in myopathies such as Duchenne Muscular Dystrophy (DMD); however, it faces considerable challenges in clinical translation, in part due to the progressive nature of the disease. Here we tested the ability of JA16 monoclonal antibody-mediated myostatin blockade to improve the dystrophic phenotype in newborn *mdx* mice (an animal model of DMD). Myostatin inhibition led to significant increases in muscle size, fiber number and cross-sectional area along with increased absolute force alongside reduced post-eccentric contraction force drop and reduced serum creatine kinase. We used the Multiparametric Muscle Improvement Score (MMIS) to objectively quantitate benefits in this preclinical study and determined that the magnitude of improvements exceeded those reported using the exact same intervention in older *mdx* mice treated for the same duration. This study demonstrates an age-dependent aspect of this intervention and suggests that earlier interventions may provide greater therapeutic benefits.

## 1. Introduction

**Duchenne Muscular Dystrophy (DMD).** DMD is a fatal, genetic disease caused by mutations in the DMD gene leading to quantitative and qualitative defects in the expression of the dystrophin protein [1,2,3]. Dystrophin is a member of the spectrin superfamily, which includes the spectrins, α-actinins and three close relatives of dystrophin, the chromosome 6-encoded dystrophin-related protein (DRP) also called utrophin [3,4,5,6], DRP 2 and dystrobrevin [7,8,9,10]. Dystrophin is associated with the membrane-bound dystroglycan/sarcoglycan complex (D/SGC) which itself is part of a larger complex of evolutionarily conserved proteins associated with dystrophin, including nNOS, the syntrophins, dystrobrevin and utrophin/DRP [11,12,13]. Mutations in the genes encoding various members of the complex and binding proteins disrupt sarcolemmal integrity and result in a variety of X-linked and limb girdle muscular dystrophies [11,14,15,16].

**DMD: Therapeutic Landscape.** Attempts at delivering the missing gene product dystrophin to DMD muscle by cell transplantation (myoblast/stem cell therapy) [17,18,19], gene editing [20,21,22,23,24,25,26,27] or delivering an internally deleted dystrophin construct via viral vectors (i.e., AAV micro dystrophin gene therapy) [28,29,30,31,32,33] face technical problems such as delivery, immune rejection and toxicity [33,34,35]. The Food and Drug Administration (FDA) has currently approved splice skipping oligos targeting Exons 51, 53 and 45 for the ca. 30% patients of DMD with mutations amenable to skipping; however, the clinical benefits are limited [36,37]. Steroids (e.g., Prednisone, Deflazacort and Vamorolone) and the histone deacetylase inhibitor Givinostat are useful in the management of DMD as they reduce inflammation and slow disease progression. However, they are not specific for the disease, and chronic use of steroids is known to be associated with multiple systemic (e.g., behavioral, metabolic, bone) side effects [38]. The FDA has approved adeno-associated virus (AAV)-mediated micro dystrophin gene therapy in the United States; however, its efficacy is unclear. Because patients do not express dystrophin, all dystrophin-based approaches face a fundamental limitation, since patients’ immune system recognizes dystrophin as foreign [39]. While the median life expectancy of DMD patients has improved over the last few decades [40], the disease course remains inexorably associated with debilitating loss of muscle mass and strength, especially as patients grow older. Given the importance of muscle mass and strength for patients, attempts have been made to increase muscle mass using a variety of dystrophin-independent approaches including G-CSF [41] and the Myostatin/Activin pathway (c.f. below) in DMD patients, with limited success. Thus, there remains a great unmet clinical need, since currently, there is no safe and effective therapy for all DMD patients.

**Myostatin.** Myostatin, an evolutionarily conserved member of the TGF-β superfamily, is a negative regulator of muscle growth [42,43,44,45,46]. Naturally existing “double-muscled” cattle with ca. 20% increased muscle mass were first bred over 200 years ago and are known to have myostatin gene mutations that lead to their increased musculature. Myostatin mutations leading to increased musculature have been described in several animal species including mice, dogs and humans [47]. As with other ligands in this superfamily, myostatin has a ca. 26 kDa inactive N-terminal, a tetrapeptide cleavage site and a ca. 12 kDa active C-terminal (mature) region. It is thought that after processing, mature myostatin binds the Myostatin/Activin (ActRII A/B) receptors, promotes heterodimerization with the ALK4/5 receptors, and phosphorylates SMAD2 and SMAD3, promoting their translocation to the nucleus and ultimately leading to modulation (inhibition) of transcriptional changes associated with myogenesis.

This pathway is of translational relevance, as targeting the Myostatin/Activin pathway has been suggested as a therapeutic strategy to increase muscle mass in several diseases including myopathies (e.g., DMD) and metabolic disorders [48,49]. However, despite encouraging preclinical animal studies [50,51,52,53], clinical trials targeting myostatin/activin inhibition have been disappointing [49,54]. While a major focus for drug development has been to increase the selectivity and/or efficacy and hence the therapeutic index of the intervention, there is a relative paucity of information regarding potential differences in outcomes of these interventions based on early versus late age of treatment initiation.

Here, we tested the hypothesis that early-age JA16 monoclonal antibody-mediated myostatin blockade in newborn *mdx* mice would improve the dystrophic phenotype to a greater extent than that seen in older *mdx* mice. Our data demonstrate an age-dependent effect of myostatin blockade on improving the dystrophic phenotype when compared to using this strategy in older mice [55].

## 2. Results

We have previously demonstrated that JA16 monoclonal antibody-mediated myostatin blockade resulted in an improvement in the dystrophic phenotype in 1-month-old *mdx* mice [55]. Since 1-month old mice are considered at the onset of sexual maturity, we conducted the current studies in younger immature animals that (a) represent the age range more relevant for younger DMD patients and (b) determine if the improvements could be further enhanced by treating earlier (and hence optimize the timing of intervention). We treated neonatal *mdx* mice with the exact same therapeutic regimen using JA 16 monoclonal antibody-mediated myostatin blockade.

**Figure 1:**
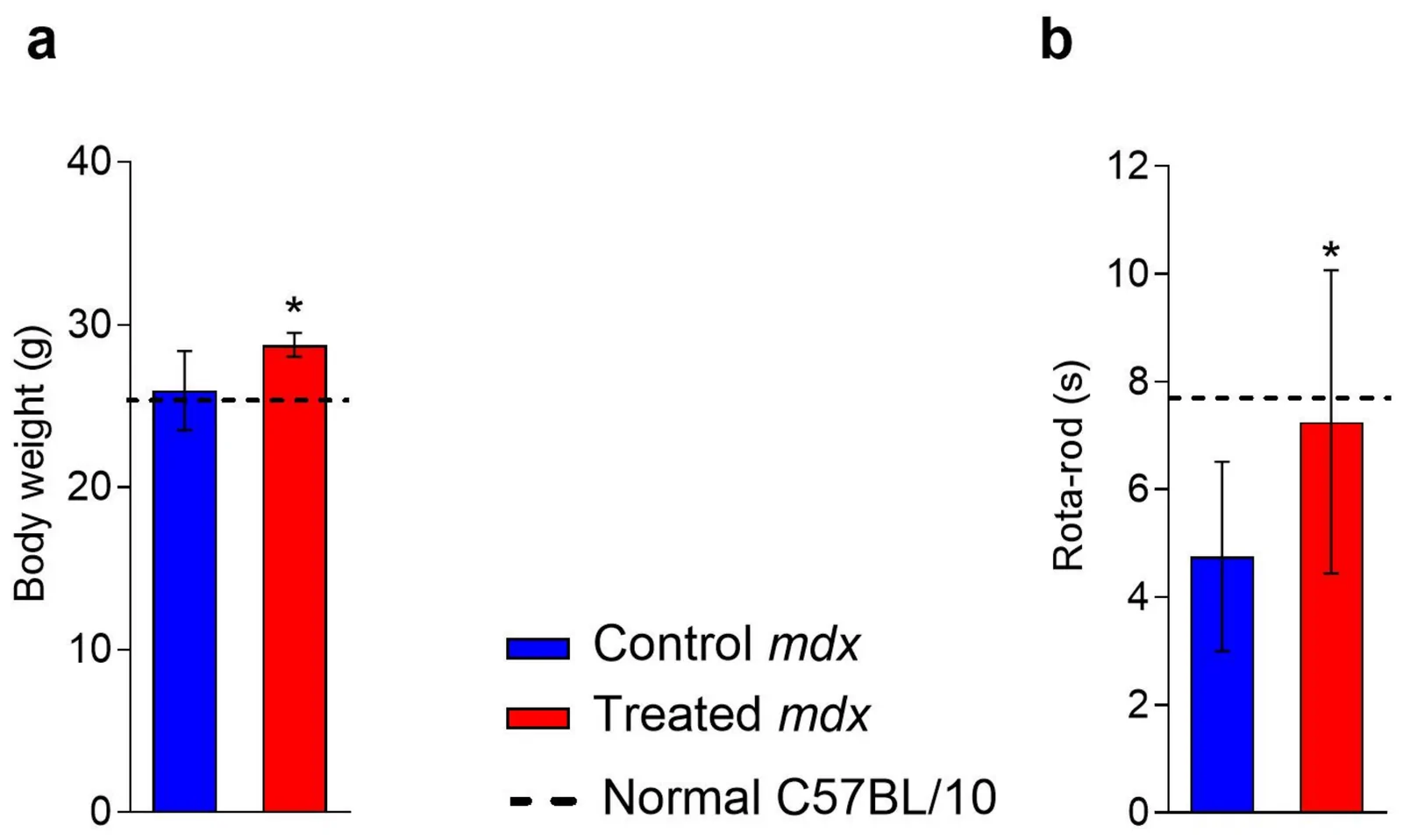
**Effects of myostatin blockade in vivo.** (**a**) Significantly increased body weight (treated *mdx*: 28.8 ± 2.1 g, n = 8; control *mdx*: 25.9 ± 2.4 g, n = 8; *p* = 0.025); Normal C57BL/10: (26.1 ± 2.8, n = 7) and (**b**) rota rod endurance time (treated *mdx*: 7.3 ± 4.8 s, n = 8; control *mdx*: 4.8 ± 1.8 s, n = 8; *p* = 0.051); normal C57BL/10: (7.8 ± 1.4 s, n = 8) was noted in *mdx* mice that had been treated with antibody-mediated myostatin blockade compared to control *mdx* mice. Normal C57BL/10 values are provided for comparison.

**Effects of myostatin blockade in vivo.** After 3 months of treatment, mice were weighed and subjected to rota rod testing in vivo. As shown in Figure 1, treated *mdx* mice had significantly increased body weights and could remain on the rotating rod for a significantly longer period of time compared to control untreated *mdx* mice.

**Figure 2:**
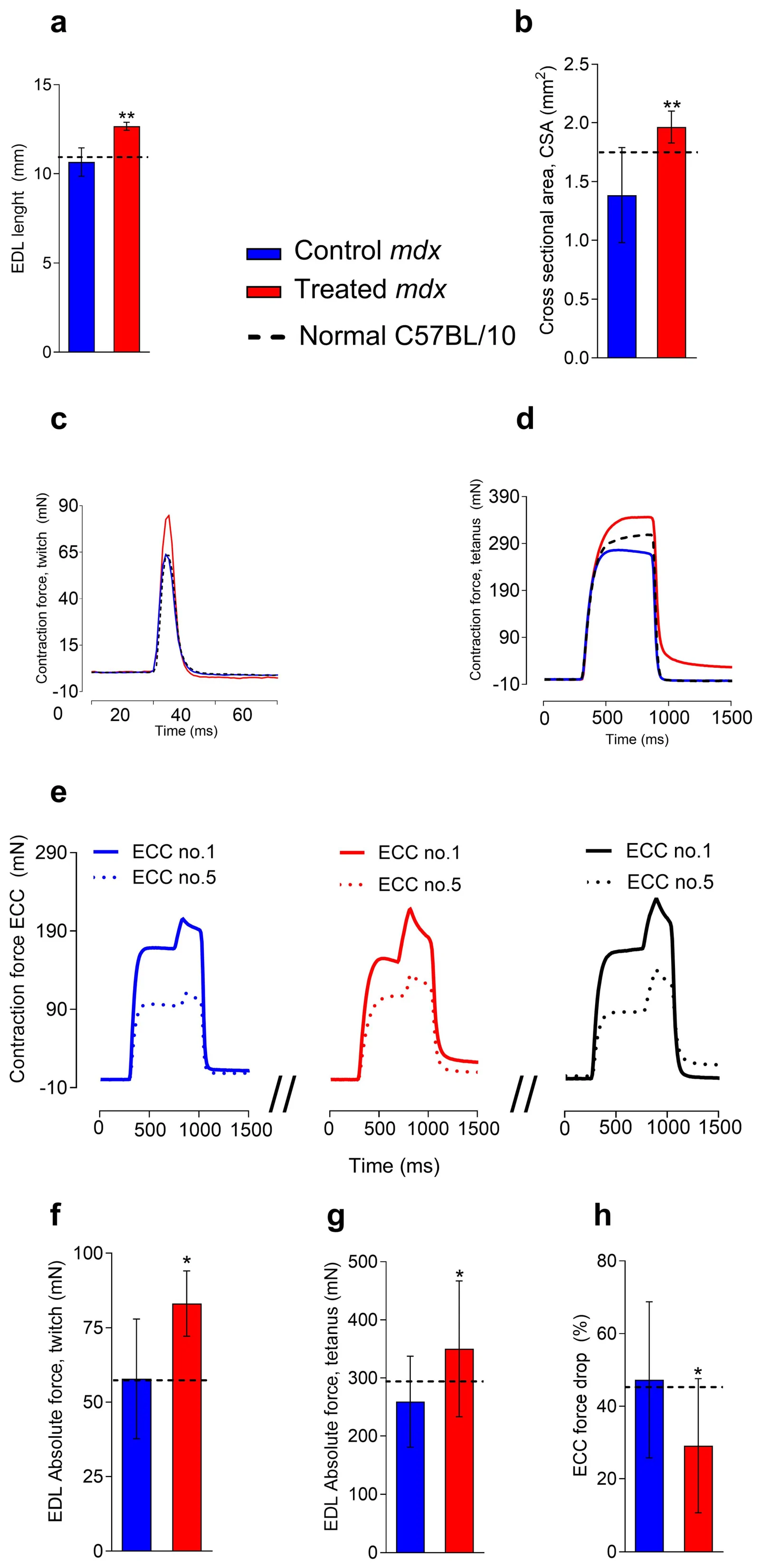
**Increase in muscle size and strength by myostatin blockade.** (**a**) Significantly increased EDL length (treated *mdx*: 12.7 ± 0.8 mm, n = 12; control *mdx*: 10.7 ± 0.8 mm, n = 12; *p* < 0.0001). Normal C57BL/10 (11.2 ± 0.6 mm, n = 12) and (**b**) cross-sectional area (treated *mdx*: 2.0 ± 0.5 mm^2^, n = 12; control *mdx*: 1.4 ± 0.4 mm^2^, n = 12; *p* = 0.0038); normal C57BL/10 (1.7 ± 0.5 mm^2^, n = 12), was noted in *mdx* mice that had been treated with antibody-mediated myostatin blockade compared to control mdx mice. Representative traces of EDL muscle twitch, tetanus and ECC force drop are shown in (**c**–**e**) panels. Significantly improved (**f**) EDL twitch force (treated *mdx*: 83.1 ± 38.1 mN, n = 12; control *mdx*: 57.8 ± 20.1 mN, n = 12; *p* = 0.053); normal C57BL/10 56.2 ± 11.5 mN, n = 12, (**g**) EDL tetanic force (treated *mdx*: 350.1 ± 116.8 mN, n = 12; control *mdx*: 259.2 ± 78.4 mN, n = 12, *p* = 0.035); normal C57BL/10 (299.5 ± 113.4 mN, n = 12) and (**h**) improved percentage force drop following ECC (treated *mdx*: 29.1 ± 18.4%, n = 12; control *mdx*: 47.3 ± 21.5%, n = 12; *p* = 0.037); normal C57BL/10 (45.3 ± 17.5%, n = 12) was noted in *mdx* mice that had been treated with antibody-mediated myostatin blockade compared to control *mdx* mice.

**Effects of myostatin blockade on contractile and morphometric properties of individual muscles.** To determine the effect of myostatin blockade on individual muscles, mice were euthanized and the extensor digitorum longus (EDL) muscle dissected and analyzed. Consistent with our previous study [53], detailed analyses showed that EDL muscles from treated *mdx* mice had significantly increased length and cross-sectional area (CSA) at the end of treatment (Table 1 and Figure 2). Quantification of physiological properties of the EDL muscles demonstrated that the absolute force generated during twitch and tetanus contractions were significantly increased in treated *mdx* mice (Figure 2). The specific forces were not increased (Table 1) suggesting that the force changes were commensurate with the increased muscle size. Additionally, there was a significant reduction (improvement) in the post-eccentric contraction (ECC) force drop percentage in treated *mdx* mice.

**Figure 3:**
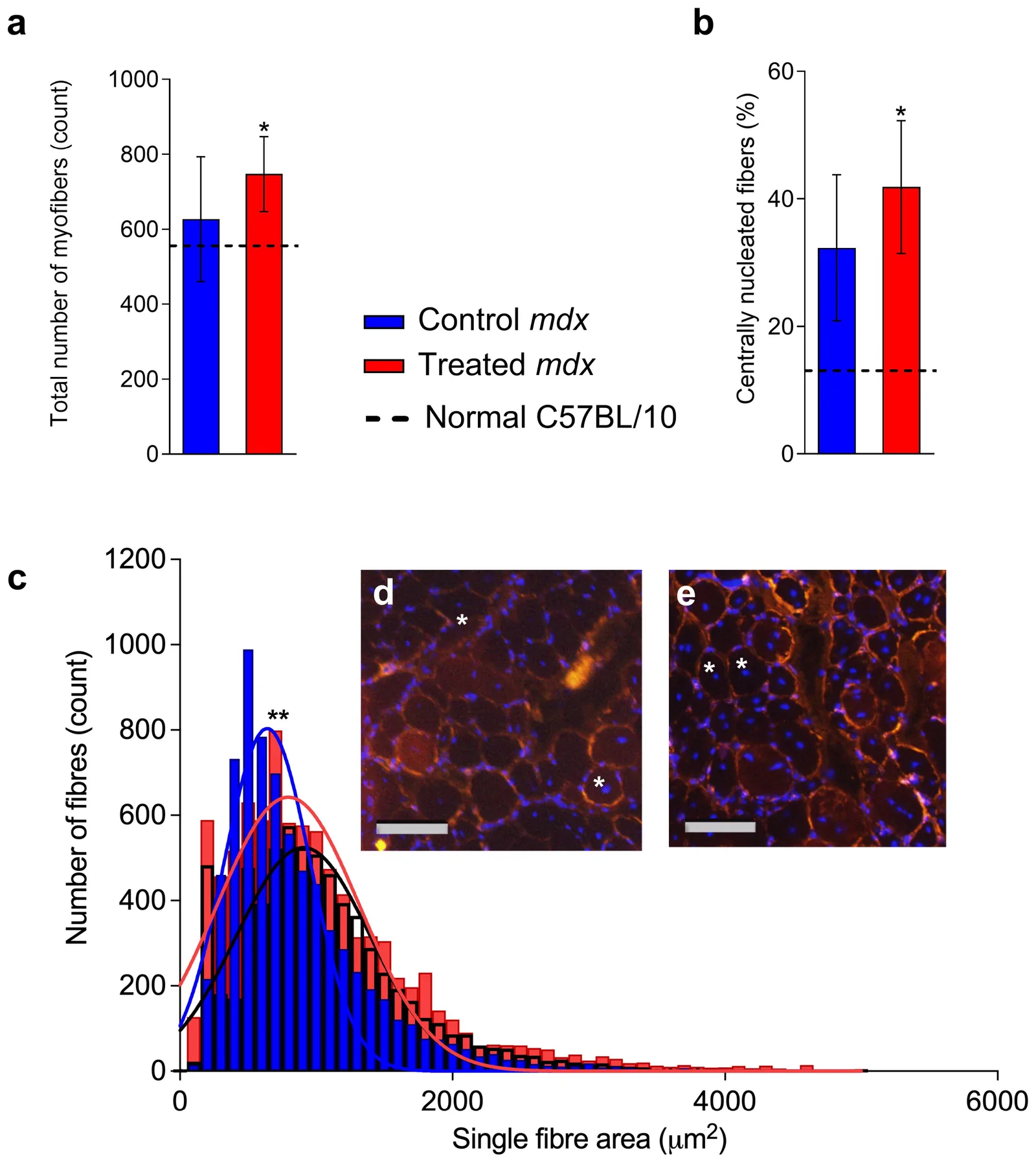
**Effects of myostatin blockade on morphometric properties of EDL muscle.** Significantly increased (**a**) number of myofibers (treated *mdx*: 732.9 ± 81.3, n = 12; control *mdx*: 620.4 ± 183.3, n = 12, *p* = 0.043); normal C57BL/10 (566.2 ± 136.8, n = 12) in treated versus control *mdx* mice, (**b**) CNF percentage (treated *mdx*: 41.9 ± 9.1%, n = 12; control *mdx*: 32.3 ± 11.5%, n = 12, *p* = 0.044); normal C57BL/10 (12.2 ± 3.9%, n = 12) and (**c**) single fiber area (treated *mdx*: 1020.3 ± 804.8 μm^2^, n = 8968; control *mdx*: 825.4 ± 574.3 μm^2^, n = 7336; *p* < 0.001); normal C57BL/10 (1025 ± 659.3 μm^2^, n = 6794) was noted in *mdx* mice that had been treated with antibody-mediated myostatin blockade compared to control *mdx* mice. Panels (**d**,**e**) show representative sections of control and treated *mdx* EDL muscles, respectively, with asterisks marking CNFs. Scale bar 100 μm.

Morphometric analyses of the EDL muscles were undertaken to quantify changes in muscle at the single fiber level, resulting from myostatin blockade. Figure 3 revealed significant increases in the number of fibers wasnoted, as well as an increase in the percentage of fibers with centrally nucleated fibers (CNFs) (Figure 3) in the EDL of treated versus control *mdx* mice.

**Figure 4:**
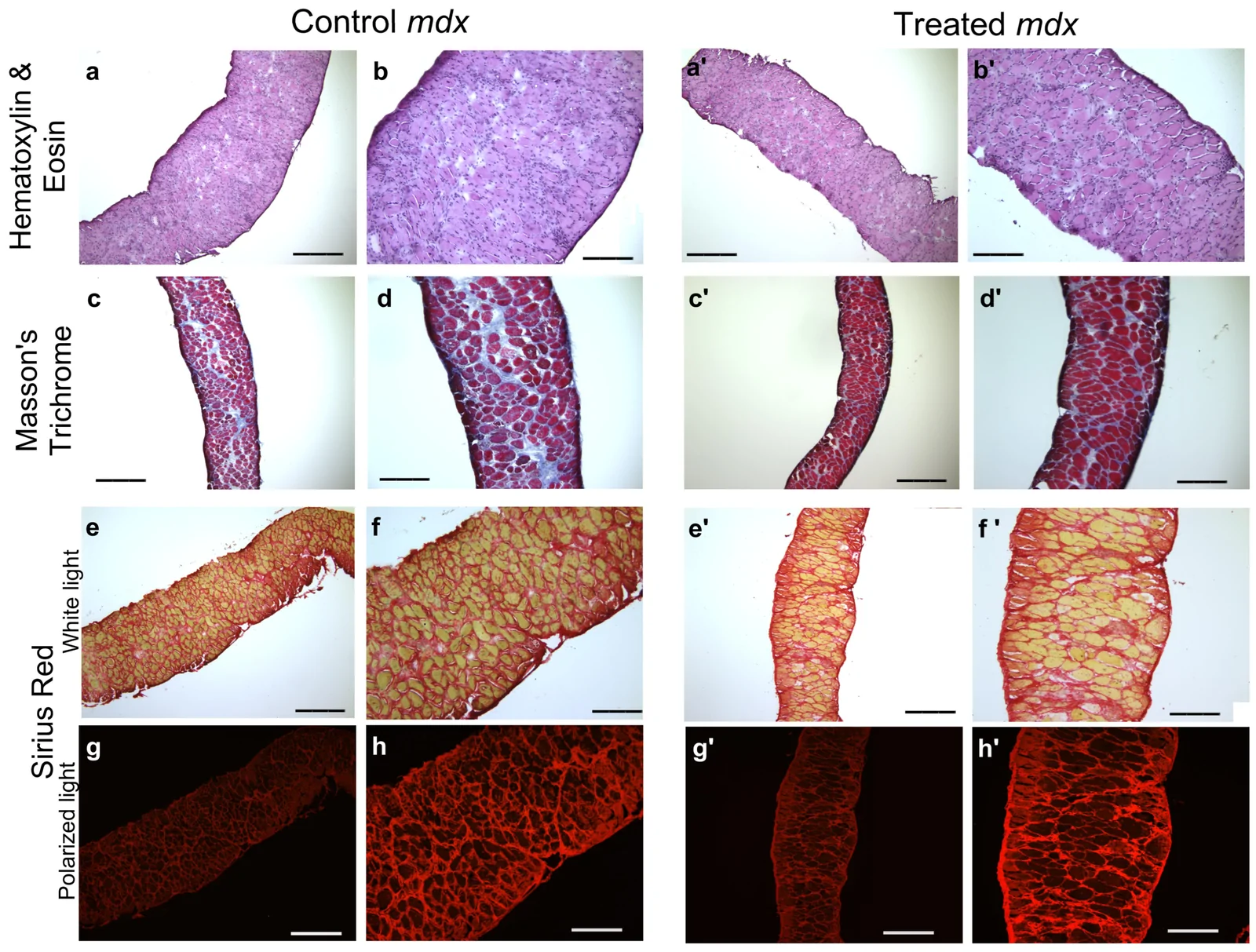
**Effects of myostatin blockade on diaphragm muscle morphology.** No significant morphological changes were noted in sections of diaphragm muscle of treated versus control *mdx* mice. Panels (**a**,**b**) show low- and high-power views of sections stained with H&E from controls, while panels (**a′**,**b′**) show low- and high-power views of sections from treated *mdx* mice. Panels (**c**,**d**) show low- and high-power views of sections stained with Masson’s Trichrome from controls, while panels (**c′**,**d′**) show low- and high-power views of sections from treated *mdx* mice. Panels (**e**,**f**) show low- and high-power views of sections stained with Sirius Red and examined with white light from controls, while panels (**e′**,**f′**) show low- and high-power views of sections stained from treated *mdx* mice. Panels (**g**,**h**) show low- and high-power views of sections stained with Sirius Red and examined with polarized light from controls, while panels (**g′**,**h′**) show low- and high-power views of sections stained from treated *mdx* mice. Magnification and Scale Bars: Panels (**a**,**c**,**e**,**g**,**a′**,**c′**,**e′**,**g′**) are 10× and scale bar is 100 μm. Panels (**b**,**d**,**f**,**h**,**b′**,**d′**,**f′**,**h′**) are 20× and scale bar is 150 μm.

To further explore and correlate the improvement in muscle function to morphological changes, we analyzed cryosections of diaphragm muscles from control and treated mdx mice using hematoxylin and eosin (H&E), Masson’s trichrome and Sirius red staining; however, no significant histological differences could be noted between control and treated *mdx* mice (Figure 4).

**Figure 5:**
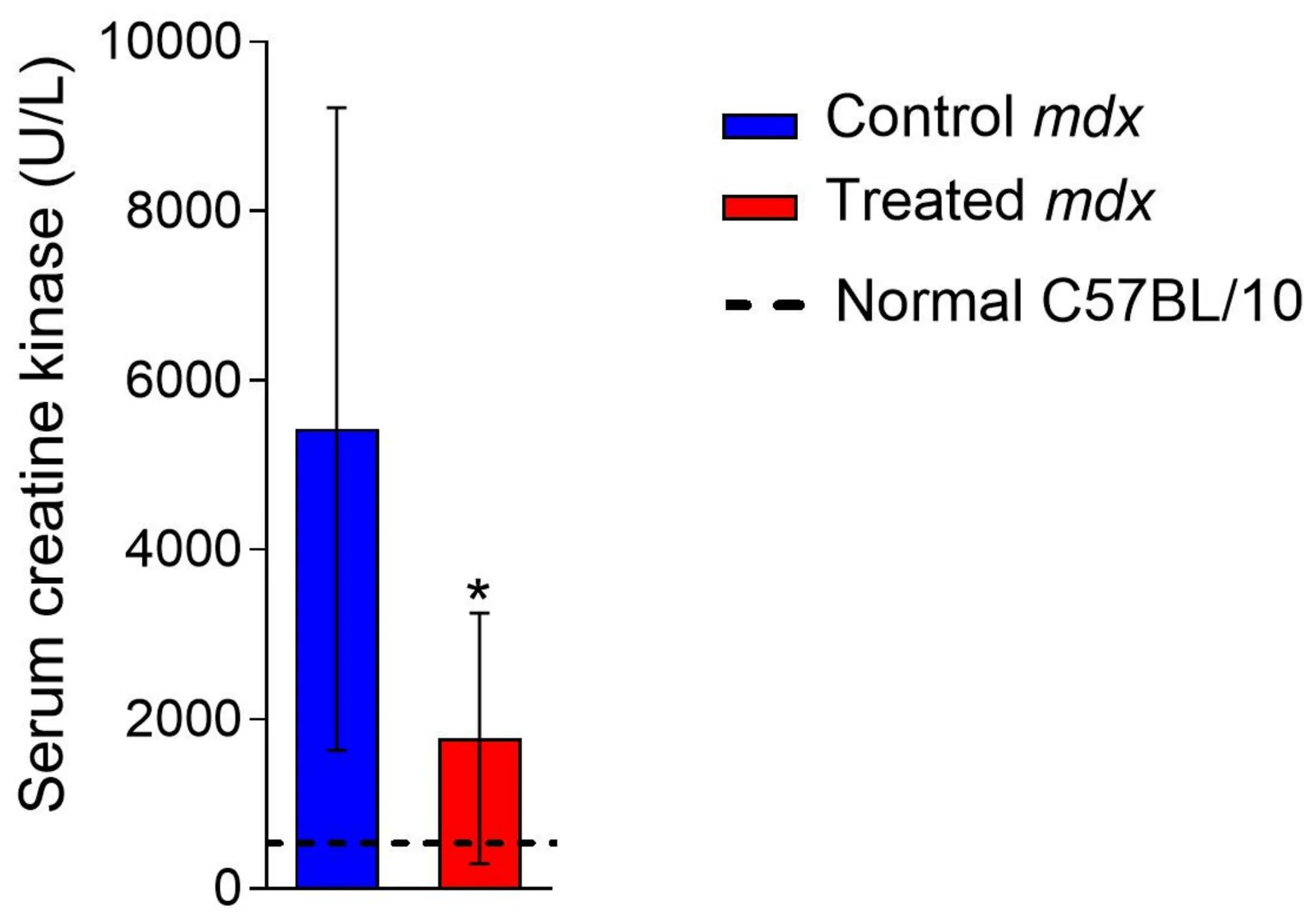
**Reduction in serum CK by myostatin blockade.** Significant reduction in serum CK was noted in treated compared to control *mdx* mice (control *mdx*: 5429 ± 3794 U/L, n = 7; treated *mdx*: 1772 ± 1478 U/L, n = 7; *p* = 0.045; normal C57BL/10,480.3 ± 227.5 U/L, n = 5).

**Effects of myostatin blockade on muscle and serum biochemical properties.** Consistent with the lack of morphological differences, diaphragm muscle hydroxyproline content was not significantly changed (treated *mdx*: 3.3 ± 0.8 µg/mg, n = 7; control *mdx*: 2.5 ± 0.5 µg/mg, n = 7; *p* = 0.080; normal C57BL/10: 1.7 ± 0.4 µg/mg, n = 7). To determine if the myostatin blockade was accompanied by changes in circulating biomarkers of muscle damage, we analyzed blood taken at the end of the trial and found that serum creatine kinase (CK) was significantly decreased in treated versus control *mdx* mice (Figure 5). Together, these results demonstrate that myostatin blockade initiated in neonatal *mdx* mice (a) resulted in an improvement but not a complete rescue of the dystrophic phenotype and (b) exceed improvements previously noted by us using the same treatment regimen in older *mdx* mice, demonstrating the age-dependent effects of myostatin blockade on the dystrophic phenotype.

To more precisely compare and quantify the differences in the current study (with an early initiation of treatment) with our previous study (with initiation of treatment at a later period) [55], we quantified the multiparametric muscle improvement score (MMIS) [56] for the preclinical trials (Table 2).

The current study had a score of 23 compared to the score obtained using a later onset of intervention which scored 21, suggesting that the neonatal intervention was more effective at improving the dystrophic phenotype.

## 3. Discussion

Myostatin/Activin is well recognized to be a promising “targetable” pathway for increasing muscle mass in disorders associated with muscle wasting, such as DMD. Despite promising results achieved by targeting this pathway in animal studies, the data from clinical trials demonstrate the need to improve and optimize this strategy [49,54,57]. Indeed, ongoing efforts have yielded novel reagents (e.g., ligand traps) targeting this pathway with greater efficacy and for broader indications (e.g., bimagrumab for metabolic disorders) [53,58,59]. Despite the well-documented progressive nature of muscle damage, wasting and fibro-fatty degeneration in DMD [1], there is a relative paucity of information regarding changes in the degree of improvement based on the age of initiation of myostatin-blockade treatment.

Here we demonstrate an age-dependent effect of antibody mediated myostatin blockade on the dystrophic phenotype. In this study, we used the exact same treatment regimen (dose, duration, myostatin blocking antibodies and analytical methodology) as our previous study [55]; however, we initiated the treatment at an earlier (neonatal) stage. Systemic delivery of myostatin-blocking antibodies led to improvements in the in vivo (Figure 1) and ex vivo parameters of muscle function (Figure 2). Myostatin blockade also led to increased muscle size, fiber number, CSA and CNFs (Figure 3). These changes were unaccompanied by improvements in the morphological properties (Figure 4) or hydroxyproline content of treated dystrophic diaphragm muscle, suggesting the treatment did not completely reverse dystrophic changes. The improvements were, however, accompanied by decreased serum CK in treated mice, signifying improvement in clinically important biochemical parameters (Figure 5).

It is noteworthy that JA16 antibody-mediated myostatin blockade led to increased muscle size and CNFs, which are consistent with some of the proposed role(s) of myostatin in modulating muscle stem cells and muscle size [44,60,61,62,63]. While the exact mechanism(s) by which myostatin-blocking antibodies led to protection against ECC damage and CK leakage are yet to be determined, it is tempting to suggest this may be related to the treated mice having to use a reduced fraction of maximal force output of the increased muscle mass for their daily activities, hence being able to better resist contractile stress-induced damage. Indeed, CK levels have been reported to be elevated upon a force generation challenge in untrained individuals [64]. We noted an increase in absolute but not specific force, suggesting that while the increase in muscle strength was commensurate with increased muscle size, some degree of contractile dysfunction persisted. Additionally, CNF proportion was not reduced. These findings are not unexpected given the natural history of the *mdx* mice, and the antibody myostatin blockade does not correct the primary defect, i.e., dystrophin deficiency. These data suggest a complex phenotype in the treated *mdx* mouse muscle consisting of reduced membrane leakage and susceptibility to lengthening damage (evidenced by reduced CK and post-ECC force drop) accompanied by increased muscle size and ongoing regeneration (evidenced by increased CSA and CNFs). Taken together, these data largely confirm and extend previous studies that have targeted the Myostatin/Activin pathway for increasing muscle mass in vivo, using a variety of genetic and pharmacological means [47,49].

The age-dependent effects of myostatin blockade on the dystrophic phenotype reported here exemplify the utility of comparative studies for helping improve the efficacy of potentially therapeutic interventions. This report also suggests the need for improving/developing standardized measurements and objective methodology for reporting outcomes to increase the information content of preclinical studies [65,66,67], exemplified by those available at the TREAT-NMD consortium (https://www.treat-nmd.org), the SHIRPA tool [68] and the MMIS [56]. We utilized MMIS which provides pre-determined weighted scores for outcome measures that are widely used in the field and hence allowed us to objectively score the current study and compare it with our previous study using the same treatment regimen but with a later age of initiation. The 34-point MMIS scale provides a single numerical score based on assigning weighted improvement points for ten parameters (e.g., specific tetanic force, decreased serum CK), with a maximum achievable value of 34 [56], and is reminiscent of the widely used 34-point North Star Ambulatory Assessment (NSAA) scale used for assessing trials in DMD [69,70]. The neonatal initiation reported in this study had a higher score (23) compared to that obtained using a later onset of intervention (21) using the multiparametric MMIS (Table 2) scoring method. Consistent with our findings, a previous report using PF-354 (a different anti-myostatin antibody) restored the functional capacity of diaphragm strips to control levels when treatment was initiated early, but not in the later stages of disease progression [71]. Additionally, inducible utrophin transgene expression demonstrated a differential improvement of dystrophic phenotype depending on the timepoint at which transgene expression was initiated [72].

Natural history studies of DMD patients along with inducible utrophin transgenic mouse data and data from antibody-mediated myostatin blockade support the notion that therapeutic interventions for DMD, such as the approach described here, would be more effective if initiated early in the disease. The recent expansion of the recommended uniform screening panel (RUSP) in the United States to include DMD should allow treatment to be initiated in the neonatal period in a larger number of patients and hence be more effective. Based on the expanded newborn screening guidelines, it is likely that in the future diagnosis at birth (rather than at ca. 4–5 years, as is the case currently) will become the norm for DMD diagnosis: we suggest that initiation of treatment in the neonatal period as described in this study, rather than later in life, may represent a clinically relevant timepoint for improving the therapeutic efficacy of interventions.

## 4. Materials and Methods

### 4.1. Mice and Treatment

Experiments were performed in neonatal (5–7-day-old) male *mdx* mice (C57BL/10ScSn-Dmd mdx/J) and age-matched control mice (C57BL/10Sc-Sn). Animals were obtained from the Jackson Laboratory (Bar Harbor, ME, USA) and were bred in the animal facility at the University of Pennsylvania. Treated mdx mice were injected intraperitoneally (i.p.) once a week with a purified blocking anti-myostatin mouse monoclonal antibody (JA16) obtained from Wyeth Research, Cambridge, MA, USA, concentration 8.4 mg/mL dose 60 mg/kg, while control *mdx* mice were injected i.p. once a week with the same volume of phosphate-buffered saline (PBS) alone, as a placebo. A group of age-matched normal mice (normal C57BL/10) were used as non-treated controls. All animal experiments were conducted in accordance with institutional guidelines and were approved by the Institutional Animal Care and Use Committee (IACUC) of the University of Pennsylvania.

### 4.2. Physiological Analysis of Muscle Function

Muscle function was analyzed using in vivo and ex vivo measurements, including rota rod performance, muscle strength (isometric absolute and specific force) and susceptibility to damage due to lengthening contractions (post-ECC force drop). The design of the apparatus and tests have been described in detail previously [53,67], as well in https://www.treat-nmd.org/wp-content/uploads/2023/07/DMD_M.1.2.002.pdf (accessed on 1 August 2026). Rota rod performance was assayed using a custom-made rota rod apparatus. The apparatus consists of a 6 mm diameter hollow plastic rod attached horizontally to a DC motor (Appropriate Technical Resources, Laurel, MD, USA), calibrated to rotate at 18 rpm and fixed at a 0.3 m height above the bench. Mice were scored for their ability to grip and hang by their forelimbs onto the slowly rotating plastic rod.

Isometric twitch and tetanic force, as well as post-ECC force drop, were measured on isolated murine EDL muscle using a custom-built apparatus. Isolated EDL muscles were dissected, weighed, tendons tied using 5–0 sutures, and the muscles transferred to an organ bath containing constantly oxygenated (gas combination: 5% CO_2_, 95% O_2_) Ringer solution maintained at 24 °C. Muscles were attached to a Grass FT 03 force transducer at one end and a step-motor-driven length controller at the other end. The muscle preparation was stimulated using a Grass S48 stimulator to develop an electrical field between two platinum electrodes placed longitudinally alongside the muscle. Muscle length was adjusted to achieve optimal twitch and tetanus and the optimal length (L0) was measured. For ECC, muscles were subjected to 5 tetanic stimuli at a total duration of 700 ms each (500 ms of isometric phase and 200 ms eccentric phase) with the muscle being lengthened by 10% L0 at a velocity of 0.5 Lo/s during the contraction. The percentage drop in force generation between the first and fifth tetanus was calculated from the isometric phase of the standard ECC protocol [55,73].

### 4.3. Morphometric Analysis

Muscles were flash-frozen after physiological analyses in isopentane cooled in liquid nitrogen. Serial frozen cryosections (8–12 μm) were cut using a cryostat (HM 500 Cryostat, Zeiss, Oberchosen, Germany). Sections were transferred to Superfrost Plus electrostatically charged slides (Menzel-Glaeser, Braunschweig, Germany), fixed for 5 min in 100% ice-cold methanol and stored in airtight containers. EDL muscle section slides were labeled with laminin antibodies, DNA-binding dye Hoechst 33,825 and processed for immunofluorescence (IF) using an Olympus BX51 System microscope with Magnafire camera. Fiber counting as well as single fiber area measurements were made at the mid-belly area of the muscle using Scion image 4.02/Image J area measurement programs. All myofibers (n = 23,286) in all three groups of physiologically examined muscles (n = 36) were photographed, counted and measured to determine the muscle cross-sectional area (CSA), single fiber area and the proportion of CNFs. Morphological analyses were performed on sections of the diaphragm muscle using a combination of H&E, Masson’s Trichrome and Sirius red staining.

### 4.4. Biochemical Analysis

Approximately 100 μL aliquots of tail vein blood were collected. Serum was separated by centrifugation and serum CK measured using a standard colorimetric assay (Stanbio Laboratory, Boerne, TX, USA), using a Cary 50 Bio Spectrophotometer (Varian Inc., Paolo Alto, CA, USA). Measurement of hydroxyproline content was performed on frozen diaphragm muscle by AAA Laboratory, Mercer Island, WA, USA.

### 4.5. Statistical Analysis

Numerical data were presented as the mean values ± standard deviation. Comparisons between the two examined groups (treated *mdx* and control *mdx*) were performed using a two-tailed Student *t*-test. Statistical significance was shown with *p* values (*p* ≤ 0.05). For graphic presentation, the following system was used throughout this manuscript: treated *mdx* (red color), control *mdx* (blue color) and normal C57BL/10 (black dashed line) and * or ** for statistically significant differences.

## 5. Patents

Tejvir S. Khurana is a co-inventor on US Patent Number 8,710,202 “Isolated nucleic acid molecule encoding an antibody that reduces GDF-8 activity” related to this work.

## Figures and Tables

**Table 1:** Contractile and morphometric properties of EDL in *mdx* and C57BL/10 mice.

Parameter	Mice		
	Control	Treated	Normal
	*mdx*	*mdx*	C57BL/10
Twitch			
Absolute force (mN)	57.8 ± 20.1	83.1 ± 38.1 (*p* = 0.053)	56.2 ± 11.5
Specific force (mN/mm^2^)	44.8 ± 21.4	42.1 ± 13.7 (*p* = 0.709)	36.1 ± 15.1
Tetanus			
Absolute force (mN)	259.2 ± 78.4	350.1 ± 116.8 (*p* = 0.035)	299.5 ± 113.4
Specific force (mN/mm^2^)	200.4 ± 81.8	181.1 ± 52.9 (*p* = 0.500)	198.9 ± 107.3
ECC force drop (%)	47.3 ± 21.5	29.1 ± 18.4 (*p* = 0.037)	45.3 ± 17.5
EDL weight (mg)	13.6 ± 2.5	15.0 ± 1.7 (*p* = 0.134)	14.7 ± 2.9
EDL length (mm)	10.7 ± 0.8	12.7 ± 0.8 (*p* < 0.0001)	11.2 ± 0.6
EDL CSA (mm^2^)	1.4 ± 0.4	2.0 ± 0.5 (*p* = 0.0038)	1.7 ± 0.5
Myofibers (count)	620.4 ± 183.3	732.9 ± 81.3 (*p* = 0.043)	566.2 ± 136.8
Myofibers (μm^2^)	825.4 ± 574.3	1020.3 ± 804.8 (*p* < 0.001)	1025 ± 659.3
CNF (%)	32.3 ± 11.5	41.9 ± 9.1 (*p* = 0.044)	12.2 ± 3.9
Other muscle groups			
Tibialis anterior weight (mg)	56.4 ± 8.81	65.9 ± 5.4 (*p* = 0.004)	42.3 ± 5.0
Quadriceps weight (mg)	217.6 ± 21.0	266.7 ± 54.6 (*p* = 0.008)	193. 7 ± 20.9
Gastrocnemius weight (mg)	170.2 ± 28.4	212.4 ± 28.1 (*p* = 0.001)	152.3 ± 20.3

**Table 2:** MMIS parameters and scores for JA16 antibody-mediated myostatin-blockade strategies in *mdx* mice.

Mice	*mdx*	*mdx*
Treatment	Antibody Bogdanovich et al. 2002 (Nature) [55]	Antibody Bogdanovich et al. 2026 (Muscles)
Body weight (g)	1	1
Muscle weights (mg)	2	2
Absolute force, twitch (mN)	1	1
Specific force, twitch (mN/mm^2^)	0	0
Absolute force, tetanus (mN)	4	4
Specific force, tetanus (mN/mm^2^)	0	0
Eccentric contractions	0	5
Centrally nucleated fibers	5	5
Loss of fibrotic changes	3	0
Decreased CK value	5	5
**Total score**	**21**	**23**

## Data Availability

The raw data supporting the conclusions of this article will be made available by the authors on request.

## Abbreviations

The following abbreviations are used in this manuscript:

DMD	Duchenne Muscular Dystrophy
MMIS	Multiparametric Muscle Improvement Score
H&E	Hematoxylin and eosin
i.p.	Intraperitoneal
CK	Creatine kinase
CSA	Cross-sectional area
CNF	Centrally nucleated fibers
EDL	Extensor digitorum longus
ECC	Eccentric contraction
NSAA	North Star Ambulatory Assessment

## References

[1] Engel A.G., Franzini-Armstrong C. (1994). Myology.

[2] Hoffman E.P., Brown R.H., Kunkel L.M. (1987). Dystrophin: The protein product of the Duchenne muscular dystrophy locus. Cell.

[3] Koenig M., Monaco A.P., Kunkel L.M. (1988). The complete sequence of dystrophin predicts a rod-shaped cytoskeletal protein. Cell.

[4] Khurana T.S., Hoffman E.P., Kunkel L.M. (1990). Identification of a chromosome 6-encoded dystrophin-related protein. J. Biol. Chem..

[5] Love D.R., Hill D.F., Dickson G., Spurr N.K., Byth B.C., Marsden R.F., Walsh F.S., Edwards Y.H., Davies K.E. (1989). An autosomal transcript in skeletal muscle with homology to dystrophin. Nature.

[6] Tinsley J.M., Blake D.J., Roche A., Fairbrother U., Riss J., Byth B.C., Knight A.E., Kendrick J.J., Suthers G.K., Love D.R. (1992). Primary structure of dystrophin-related protein. Nature.

[7] Roberts R.G., Freeman T.C., Kendall E., Vetrie D.L., Dixon A.K., Shaw S.C., Bone Q., Bobrow M. (1996). Characterization of DRP2, a novel human dystrophin homologue. Nat. Genet..

[8] Khurana T.S., Engle E.C., Bennett R.R., Silverman G.A., Seling S., Bruns G.A., Kunkel L.M. (1994). (CA) repeat polymorphism in the chromosome 18 encoded dystrophin-like protein. Hum. Mol. Genet..

[9] Blake D.J., Nawrotzki R., Peters M.F., Froehner S.C., Davies K.E. (1996). Isoform diversity of dystrobrevin, the murine 87-kDa postsynaptic protein. J. Biol. Chem..

[10] Sadoulet-Puccio H.M., Khurana T.S., Cohen J.B., Kunkel L.M. (1996). Cloning and characterization of the human homologue of a dystrophin related phosphoprotein found at the Torpedo electric organ post-synaptic membrane. Hum. Mol. Genet..

[11] Campbell K.P. (1996). Three muscular dystrophies: Loss of cytoskeleton-extracellular matrix linkage. Cell.

[12] Ervasti J.M., Kahl S.D., Campbell K.P. (1991). Purification of dystrophin from skeletal muscle. J. Biol. Chem..

[13] Matsumura K., Ervasti J.M., Ohlendieck K., Kahl S.D., Campbell K.P. (1992). Association of dystrophin-related protein with dystrophin-associated proteins in *mdx* mouse muscle. Nature.

[14] Brown R.H., Hoffman E.P. (1988). Molecular Biology of Duchenne muscular dystrophy. Trends Neurosci..

[15] Brown R.H. (1997). Dystrophin-associated proteins and the muscular dystrophies. Annu. Rev. Med..

[16] Brown S.C., Lucy J.A. (1997). Dystrophin: Gene, Protein and Cell Biology.

[17] Gussoni E., Pavlath G.K., Lanctot A.M., Sharma K.R., Miller R.G., Steinman L., Blau H.M. (1992). Normal dystrophin transcripts detected in Duchenne muscular dystrophy patients after myoblast transplantation. Nature.

[18] Huard J., Bouchard J.P., Roy R., Malouin F., Dansereau G., Labrecque C., Albert N., Richards C.L., Lemieux B., Tremblay J.P. (1992). Human myoblast transplantation: Preliminary results of 4 cases. Muscle Nerve.

[19] Mendell J.R., Kissel J.T., Amato A.A., King W., Signore L., Prior T.W., Sahenk Z., Benson S., McAndrew P.E., Rice R. (1995). Myoblast Transfer in the Treatment of Duchenne’s Muscular Dystrophy. N. Engl. J. Med..

[20] Nik-Ahd F., Bertoni C. (2014). Ex vivo gene editing of the dystrophin gene in muscle stem cells mediated by peptide nucleic acid single stranded oligodeoxynucleotides induces stable expression of dystrophin in a mouse model for Duchenne muscular dystrophy. Stem Cells.

[21] Long C., McAnally J.R., Shelton J.M., Mireault A.A., Bassel-Duby R., Olson E.N. (2014). Prevention of muscular dystrophy in mice by CRISPR/Cas9-mediated editing of germline DNA. Science.

[22] Ousterout D.G., Kabadi A.M., Thakore P.I., Majoros W.H., Reddy T.E., Gersbach C.A. (2015). Multiplex CRISPR/Cas9-based genome editing for correction of dystrophin mutations that cause Duchenne muscular dystrophy. Nat. Commun..

[23] Long C., Amoasii L., Mireault A.A., McAnally J.R., Li H., Sanchez-Ortiz E., Bhattacharyya S., Shelton J.M., Bassel-Duby R., Olson E.N. (2016). Postnatal genome editing partially restores dystrophin expression in a mouse model of muscular dystrophy. Science.

[24] Nelson C.E., Hakim C.H., Ousterout D.G., Thakore P.I., Moreb E.A., Castellanos Rivera R.M., Madhavan S., Pan X., Ran F.A., Yan W.X. (2016). In vivo genome editing improves muscle function in a mouse model of Duchenne muscular dystrophy. Science.

[25] Tabebordbar M., Zhu K., Cheng J.K., Chew W.L., Widrick J.J., Yan W.X., Maesner C., Wu E.Y., Xiao R., Ran F.A. (2016). In vivo gene editing in dystrophic mouse muscle and muscle stem cells. Science.

[26] Young C.S., Hicks M.R., Ermolova N.V., Nakano H., Jan M., Younesi S., Karumbayaram S., Kumagai-Cresse C., Wang D., Zack J.A. (2016). A Single CRISPR-Cas9 Deletion Strategy that Targets the Majority of DMD Patients Restores Dystrophin Function in hiPSC-Derived Muscle Cells. Cell Stem Cell.

[27] Hakim C.H., Wasala N.B., Nelson C.E., Wasala L.P., Yue Y., Louderman J.A., Lessa T.B., Dai A., Zhang K., Jenkins G.J. (2018). AAV CRISPR editing rescues cardiac and muscle function for 18 months in dystrophic mice. JCI Insight.

[28] Harper S.Q., Hauser M.A., DelloRusso C., Duan D., Crawford R.W., Phelps S.F., Harper H.A., Robinson A.S., Engelhardt J.F., Brooks S.V. (2002). Modular flexibility of dystrophin: Implications for gene therapy of Duchenne muscular dystrophy. Nat. Med..

[29] Yue Y., Li Z., Harper S.Q., Davisson R.L., Chamberlain J.S., Duan D. (2003). Microdystrophin gene therapy of cardiomyopathy restores dystrophin-glycoprotein complex and improves sarcolemma integrity in the *mdx* mouse heart. Circulation.

[30] Duan D. (2018). Systemic AAV Micro-dystrophin Gene Therapy for Duchenne Muscular Dystrophy. Mol. Ther..

[31] Asher D.R., Thapa K., Dharia S.D., Khan N., Potter R.A., Rodino-Klapac L.R., Mendell J.R. (2020). Clinical development on the frontier: Gene therapy for duchenne muscular dystrophy. Expert Opin. Biol. Ther..

[32] Potter R.A., Griffin D.A., Heller K.N., Peterson E.L., Clark E.K., Mendell J.R., Rodino-Klapac L.R. (2021). Dose-Escalation Study of Systemically Delivered rAAVrh74.MHCK7.micro-dystrophin in the *mdx* Mouse Model of Duchenne Muscular Dystrophy. Hum. Gene Ther..

[33] Manini A., Abati E., Nuredini A., Corti S., Comi G.P. (2021). Adeno-Associated Virus (AAV)-Mediated Gene Therapy for Duchenne Muscular Dystrophy: The Issue of Transgene Persistence. Front. Neurol..

[34] Nance M.E., Duan D. (2015). Perspective on Adeno-Associated Virus Capsid Modification for Duchenne Muscular Dystrophy Gene Therapy. Hum. Gene Ther..

[35] Hollinger K., Chamberlain J.S. (2015). Viral vector-mediated gene therapies. Curr. Opin. Neurol..

[36] Khan N., Eliopoulos H., Han L., Kinane T.B., Lowes L.P., Mendell J.R., Gordish-Dressman H., Henricson E.K., McDonald C.M. (2019). Eteplirsen Investigators and the CINRG DNHS Investigators. Eteplirsen Treatment Attenuates Respiratory Decline in Ambulatory and Non-Ambulatory Patients with Duchenne Muscular Dystrophy. J. Neuromuscul. Dis..

[37] McDonald C.M., Shieh P.B., Abdel-Hamid H.Z., Connolly A.M., Ciafaloni E., Wagner K.R., Goemans N., Mercuri E., Khan N., Koenig E. (2021). Open-Label Evaluation of Eteplirsen in Patients with Duchenne Muscular Dystrophy Amenable to Exon 51 Skipping: PROMOVI Trial. J. Neuromuscul. Dis..

[38] Griggs R.C., Miller J.P., Greenberg C.R., Fehlings D.L., Pestronk A., Mendell J.R., Moxley R.T., King W., Kissel J.T., Cwik V. (2016). Efficacy and safety of deflazacort vs prednisone and placebo for Duchenne muscular dystrophy. Neurology.

[39] Davies K.E., Chamberlain J.S. (2019). Surrogate gene therapy for muscular dystrophy. Nat. Med..

[40] Broomfield J., Hill M., Guglieri M., Crowther M., Abrams K. (2021). Life Expectancy in Duchenne Muscular Dystrophy: Reproduced Individual Patient Data Meta-analysis. Neurology.

[41] Sienkiewicz D., Kulak W., Okurowska-Zawada B., Paszko-Patej G., Wojtkowski J., Sochon K., Kalinowska A., Okulczyk K., Sienkiewicz J., McEachern E. (2017). Efficacy and the Safety of Granulocyte Colony-Stimulating Factor Treatment in Patients with Muscular Dystrophy: A Non-Randomized Clinical Trial. Front. Neurol..

[42] McPherron A.C., Lawler A.M., Lee S.J. (1997). Regulation of skeletal muscle mass in mice by a new TGF-beta superfamily member. Nature.

[43] Lee S.J., McPherron A.C. (2001). Regulation of myostatin activity and muscle growth. Proc. Natl. Acad. Sci. USA.

[44] Thomas M., Langley B., Berry C., Sharma M., Kirk S., Bass J., Kambadur R. (2000). Myostatin, a negative regulator of muscle growth, functions by inhibiting myoblast proliferation. J. Biol. Chem..

[45] Tries R.S., Chen T., Da Vies M.V., Tomkinson K.N., Pearson A.A., Shakey Q.A., Wolfman N.M. (2001). GDF-8 propeptide binds to GDF-8 and antagonizes biological activity by inhibiting GDF-8 receptor binding. Growth Factors.

[46] Rios R., Carneiro I., Arce V.M., Devesa J. (2001). Myostatin regulates cell survival during C2C12 myogenesis. Biochem. Biophys. Res. Commun..

[47] Lee S.J. (2023). Myostatin: A Skeletal Muscle Chalone. Annu. Rev. Physiol..

[48] Khurana T.S., Davies K.E. (2003). Pharmacological strategies for muscular dystrophy. Nat. Rev. Drug Discov..

[49] Wetzlich B., Nyakundi B.B., Yang J. (2025). Therapeutic applications and challenges in myostatin inhibition for enhanced skeletal muscle mass and functions. Mol. Cell. Biochem..

[50] Akpan I., Goncalves M.D., Dhir R., Yin X., Pistilli E.E., Bogdanovich S., Khurana T.S., Ucran J., Lachey J., Ahima R.S. (2009). The effects of a soluble activin type IIB receptor on obesity and insulin sensitivity. Int. J. Obes..

[51] Pistilli E.E., Bogdanovich S., Mosqueira M., Lachey J., Seehra J., Khurana T.S. (2010). Pretreatment with a soluble activin type IIB receptor/Fc fusion protein improves hypoxia-induced muscle dysfunction. Am. J. Physiol.-Regul. Integr. Comp. Physiol..

[52] Pistilli E.E., Bogdanovich S., Goncalves M.D., Ahima R.S., Lachey J., Seehra J., Khurana T. (2011). Targeting the activin type IIB receptor to improve muscle mass and function in the *mdx* mouse model of Duchenne muscular dystrophy. Am. J. Pathol..

[53] Cadena S.M., Bogdanovich S., Khurana T.S., Pullen A., Pearsall R.S., Curran E., Faucette R., Lane J., Seehra J., Lachey J.L. (2026). ACE-031, a soluble activin type IIB receptor, increases muscle mass and strength in the common marmoset (*Callithrix jacchus*). PLoS ONE.

[54] Wagner K.R. (2020). The elusive promise of myostatin inhibition for muscular dystrophy. Curr. Opin. Neurol..

[55] Bogdanovich S., Krag T.O., Barton E.R., Morris L.D., Whittemore L.A., Ahima R.S., Khurana T.S. (2002). Functional improvement of dystrophic muscle by myostatin blockade. Nature.

[56] Bogdanovich S., Pistilli E.E. (2012). From Basic Research to Clinical Trials: Preclinical Trial Evaluation in Mouse Models, Muscular Dystrophy.

[57] Muntoni F., Byrne B.J., McMillan H.J., Ryan M.M., Wong B.L., Dukart J., Bansal A., Cosson V., Dreghici R., Guridi M. (2024). The Clinical Development of Taldefgrobep Alfa: An Anti-Myostatin Adnectin for the Treatment of Duchenne Muscular Dystrophy. Neurol. Ther..

[58] Heymsfield S.B., Coleman L.A., Miller R., Rooks D.S., Laurent D., Petricoul O., Praestgaard J., Swan T., Wade T., Perry R.G. (2021). Effect of Bimagrumab vs Placebo on Body Fat Mass Among Adults with Type 2 Diabetes and Obesity: A Phase 2 Randomized Clinical Trial. JAMA Netw. Open.

[59] Heymsfield S.B., Aronne L.J., Montgomery P., Klickstein L.B., Coleman L.A., Dole K., Mindeholm L., Spruill S., Li X., Attie K.M. (2026). Bimagrumab plus semaglutide alone or in combination for the treatment of obesity: A randomized phase 2 trial. Nat. Med..

[60] Langley B., Thomas M., Bishop A., Sharma M., Gilmour S., Kambadur R. (2002). Myostatin inhibits myoblast differentiation by down-regulating MyoD expression. J. Biol. Chem..

[61] McCroskery S., Thomas M., Maxwell L., Sharma M., Kambadur R. (2003). Myostatin negatively regulates satellite cell activation and self-renewal. J. Cell Biol..

[62] Wang Q., McPherron A.C. (2012). Myostatin inhibition induces muscle fibre hypertrophy prior to satellite cell activation. J. Physiol..

[63] Guardiola O., Lafuste P., Brunelli S., Iaconis S., Touvier T., Mourikis P., De Bock K., Lonardo E., Andolfi G., Bouche A. (2012). Cripto regulates skeletal muscle regeneration and modulates satellite cell determination by antagonizing myostatin. Proc. Natl. Acad. Sci. USA.

[64] Vincent H.K., Vincent K.R. (1997). The effect of training status on the serum creatine kinase response, soreness and muscle function following resistance exercise. Int. J. Sports Med..

[65] Grounds M.D., Radley H.G., Lynch G.S., Nagaraju K., De Luca A. (2008). Towards developing standard operating procedures for pre-clinical testing in the *mdx* mouse model of Duchenne muscular dystrophy. Neurobiol. Dis..

[66] Spurney C.F., Gordish-Dressman H., Guerron A.D., Sali A., Pandey G.S., Rawat R., Van Der Meulen J.H., Cha H.J., Pistilli E.E., Partridge T.A. (2009). Preclinical drug trials in the *mdx* mouse: Assessment of reliable and sensitive outcome measures. Muscle Nerve.

[67] Nagaraju K., Willmann R., Network T.-N., TREAT-NMD Network and the Wellstone Muscular Dystrophy Cooperative Research Network (2009). Developing standard procedures for murine and canine efficacy studies of DMD therapeutics: Report of two expert workshops on “Pre-clinical testing for Duchenne dystrophy”: Washington DC, October 27th–28th 2007 and Zurich, June 30th–July 1st 2008. Neuromuscul. Disord..

[68] Rogers D.C., Fisher E.M., Brown S.D., Peters J., Hunter A.J., Martin J.E. (1997). Behavioral and functional analysis of mouse phenotype: SHIRPA, a proposed protocol for comprehensive phenotype assessment. Mamm. Genome.

[69] Mazzone E.S., Messina S., Vasco G., Main M., Eagle M., D’Amico A., Doglio L., Politano L., Cavallaro F., Frosini S. (2009). Reliability of the North Star Ambulatory Assessment in a multicentric setting. Neuromuscul. Disord..

[70] Muntoni F., Guglieri M., Mah J.K., Wagner K.R., Brandsema J.F., Butterfield R.J., McDonald C.M., Mayhew A.G., Palmer J.P., Marraffino S. (2022). Novel approaches to analysis of the North Star Ambulatory Assessment (NSAA) in Duchenne muscular dystrophy (DMD): Observations from a phase 2 trial. PLoS ONE.

[71] Murphy K.T., Ryall J.G., Snell S.M., Nair L., Koopman R., Krasney P.A., Ibebunjo C., Holden K.S., Loria P.M., Salatto C.T. (2010). Antibody-directed myostatin inhibition improves diaphragm pathology in young but not adult dystrophic *mdx* mice. Am. J. Pathol..

[72] Squire S., Raymackers J.M., Vandebrouck C., Potter A., Tinsley J., Fisher R., Gillis J.M., Davies K.E. (2002). Prevention of pathology in *mdx* mice by expression of utrophin: Analysis using an inducible transgenic expression system. Hum. Mol. Genet..

[73] Krag T.O., Bogdanovich S., Jensen C.J., Fischer M.D., Hansen-Schwartz J., Javazon E.H., Flake A.W., Edvinsson L., Khurana T.S. (2004). Heregulin ameliorates the dystrophic phenotype in *mdx* mice. Proc. Natl. Acad. Sci. USA.

